# Special Issue “The Molecular and Cellular Pathophysiologic Mechanisms Underlying Ocular Diseases and Emerging Therapies”

**DOI:** 10.3390/ijms25042405

**Published:** 2024-02-18

**Authors:** Snježana Kaštelan

**Affiliations:** Department of Ophthalmology, Clinical Hospital Dubrava, School of Medicine, University of Zagreb, 10000 Zagreb, Croatia; snjezana.kastelan@mef.hr

Visual impairment and ophthalmic diseases represent significant global public health concerns, and their prevalence continues to rise [1,2,3]. It is important for researchers and eyecare professionals to enhance their understanding of the associated risk factors, genetic and biochemical biomarkers, as well as the cellular and molecular mechanisms underlying various eye diseases. Identifying the molecular and cellular mechanisms responsible for the pathophysiological processes of eye diseases is crucial for preventing these conditions, achieving early diagnosis, and implementing novel therapeutic strategies.

The aim of this Special Issue is to broaden knowledge of the biomarkers and biological pathways underlying the development of eye diseases and to identify molecules that may serve as treatment targets. It offers new advancements in the molecular and cellular pathogenesis of eye diseases as well as the development of preventive measures and emerging therapies. This Special Issue, titled ‘The Molecular and Cellular Pathophysiologic Mechanisms Underlying Ocular Diseases and Emerging Therapies’, of the *International Journal of Molecular Sciences* encompasses a total of eight interesting papers, comprising two original research papers, five reviews, and one case report, all presenting novel information in this expansive field of research.

This Special Issue begins with a collection of papers that investigate various aspects of the pathogenesis, prevention, clinical manifestations, and treatment options for several retinal diseases. Diabetic retinopathy (DR) is the most common eye complication in individuals with diabetes and the most prevalent microvascular complication. As a leading cause of vision loss and blindness in working-age adults worldwide, there is a growing emphasis on early prevention and the benefits of controlling modifiable risk factors [4,5,6]. Two review papers featured in this Special Issue examine the pathogenesis of DR in relation to specific dietary patterns and the supplementation of selected vitamins, microelements, and macronutrients. This knowledge has the potential to enhance our efforts in preventing DR. 

Gverović Antunica and colleagues [7] comprehensively reviewed the role of vitamin D in the pathophysiology and development of DR. Vitamin D is a crucial nutrient involved in various essential bodily functions, including immune system regulation, neuroprotection, and the modulation of inflammatory and angiogenic processes, all of which contribute to the pathophysiological mechanisms of DR [8]. Numerous studies suggest a correlation between vitamin D deficiency and an increased risk of developing DR [9,10]. While the link between vitamin D and DR is evident and clinical studies have proven the effectiveness of vitamin D supplementation in the treatment of DR, the precise underlying pathophysiological mechanisms are inadequately elucidated. Consequently, further research is imperative to gain a more profound understanding of this relationship and to determine whether vitamin D supplementation genuinely offers preventive and therapeutic benefits for individuals with DR. The close association between the protective role and impact of the Mediterranean diet, recognized as one of the healthiest dietary patterns available on DR, was investigated by Bryl et al. [11] The authors summarized and presented well-documented available data regarding the influence of the Mediterranean diet on the course of type 2 diabetes, the protective effect of certain components of this diet in the development of DR, and the potential mechanisms involved. Owing to its diverse variety of natural components, the Mediterranean diet comprises properties that include antioxidants, chemoprevention, and anti-inflammatory effects. This dietary pattern has been shown to reduce triglyceride and cholesterol levels as well as lower postprandial glycemia. It plays an important role in decreasing the risk of diabetes development and is thought to have a significant impact on DR, although research in this domain is still limited [12,13]. Given that retinopathy affects approximately one-third of individuals with diabetes, further prospective studies are essential, and the promotion of the Mediterranean diet as a healthy dietary choice needs to be widely encouraged and popularized. Zufiaurre-Seijo et al. [14] have offered interesting insights into the involvement of C2orf71/PCARE in retinal diseases. Within their review, they have provided an extensive summary of the existing knowledge regarding retinal diseases stemming from mutations in the C2orf7, RP54, and CDR genes. This comprehensive overview encompasses clinical symptoms, the mutational landscape, molecular and functional studies on photoreceptor-specific cilia, cellular and animal models, and a summary of the potential therapeutic options. In addition to reviewing the current state of knowledge, the authors have also attempted to identify gaps in the current understanding of the pathogenic mechanisms of C2orf71/PCARE and have proposed directions for future research. In a case report by Ikić Matijašević et al. [15], an interesting and educational case of systemic lupus erythematosus (SLE) with two severe complications, namely life-threatening macrophage activation syndrome (MAS) and vision-threatening retinal vasculitis (RV), have been described. Both MAS and RV are manifestations of lupus that are often inadequately recognized in their early stages, and currently, no established treatment recommendations are available. Based on the current literature, these two complications have not previously been reported in the same patient. This paper presents the course of diagnostic and treatment undertaken in a patient who responded successfully to a regimen of systemic immunosuppressants, high-dose glucocorticoids, rituximab, along with intraocular therapy that includes intravitreal bevacizumab and laser photocoagulation. Diagnosing MAS in SLE presents a significant challenge due to the overlap in symptoms, signs, and laboratory findings between the two conditions. This similarity can result in delayed diagnosis and hinder timely treatment. Furthermore, there are currently no validated criteria for diagnosing secondary hemophagocytic lymphohistiocytosis or MAS in SLE. This paper has discussed the difficulties of distinguishing MAS from SLE and offers valuable diagnostic guidelines, emphasizing the need to initiate treatment even though only a suspicion of MAS was made to prevent a fatal outcome. 

Another series of papers in this Special Issue focuses on research related to tears, including studies on the sexual dimorphism of the human lacrimal gland, dry eye disease, and tear biomarkers. Hat et al. [16] conducted an extensive investigation into the expression of androgen receptors (ARs) and estrogen receptors (ERs) within the human lacrimal gland. In this original research article, the authors analyzed tissue samples from the human lacrimal gland collected from cornea donors. This study represents the first quantitative assessment of sex steroid hormones receptor mRNA expression in the human lacrimal gland, a rare and challenging tissue sample to obtain. AR, Erα, and Erβ mRNA were identified in all samples, and their expression was measured using qPCR. Selected samples underwent immunohistochemical staining to assess receptor protein expression. The study revealed that ERα mRNA expression was significantly higher than the expression of AR and ERβ. The key finding was the prevalence of ERα mRNA in both male and female human lacrimal gland samples, suggesting that ERα may be a significant target for future research on selective hormonal therapy for dry eye disease (DED). Schlegel et al. [17] have advanced our understanding of the pathogenesis and treatment of DED. Their research provides evidence that individuals with DED exhibit distinct compositional and functional features of the ocular surface microbiome (OSM). They have also provided data on the tear proteome profile of DED patients compared to non-DED individuals. Despite the array of available treatment options, there is a current lack of specific strategies to foster a healthy ocular surface microbiome that could potentially alleviate DED. This deficiency primarily arises from limited knowledge regarding the composition of OSM and the role of its resident bacteria. The discoveries made in this study have the potential to open doors for the development of innovative interventions aimed at addressing DED. Kaštelan et al. [18] have presented intriguing findings in their review article, exploring the potential of tear biomarkers in Alzheimer’s disease (AD). It is encouraging that the available data imply that several tear biomarkers may serve as early indicators of AD, a progressive age-related neurodegenerative brain disorder that stands as the most prevalent form of dementia. AD presents a considerable diagnostic challenge that necessitates timely identification and treatment. While effective therapies for AD are currently lacking, certain medications may slow their progression [19]. The close connection between the eyes and the brain indicates that tears could offer a valuable source of AD biomarkers, even though research in this area is limited. Current data suggest that potential tear biomarkers for AD encompass lipocalin-1, lysozyme-C, lacritin, eIF4E, Aβ38, Aβ40, Aβ42, Aβ1-42, T-tau, and P-tau. Furthermore, promising candidates such as miRNAs and lactoferrin are emerging as noteworthy biomarkers for AD. Identifying these biomarkers in tears could lead to the development of cost-effective, non-invasive methods for screening, diagnosis, and monitoring of the disease. 

Finally, in a comprehensive review article by Pašalić et al. [20], current knowledge regarding the genetic and epigenetic characteristics of uveal melanoma (UM) is summarized. Molecular research has enhanced our understanding of the genetic and epigenetic mechanisms that underlie the biological behavior of UM. To enhance the treatment of UM patients, the focus should be directed toward the research of diagnostic, prognostic, and therapeutic biomarkers, as well as tumor immunogenicity and the fundamental mechanisms of tumor formation and dissemination. A more profound understanding of the molecular changes underlying UM development offers a new perspective for improved and targeted therapeutic agents that can impact specific stages of tumor development through a personalized approach. Genetic analysis has the potential to identify patients at a high risk of UM metastases, enabling more effective treatment and patient monitoring. Alterations in miRNA expression could be valuable for UM diagnosis and prognosis given the unique miRNA profiles found in melanoma cells or tissues and their association with metastasis. Advances in the molecular analysis of UM provide opportunities for a deeper understanding of melanoma pathogenesis and biological behavior, developing targeted therapeutic strategies that focus on the relevant signaling pathways and the identification of patients who will benefit most from emerging therapeutic agents.

## Conclusions and Perspectives

Overall, this Special Issue represents significant progress in unravelling the pathophysiological mechanisms of eye diseases, biomarkers, and new therapeutic options. It is intriguing that five out of the eight featured articles focus on retinal diseases, and it is noteworthy that DED is also currently a topic of considerable interest. Even though this Special Issue has enriched our understanding of the molecular and cellular pathophysiological mechanisms underlying eye diseases and innovative therapies, there are still numerous unanswered questions. We anticipate that additional, in-depth insights into the molecular aspects of eye diseases will gradually emerge in the future thanks to extensive research efforts employing highly sophisticated and advanced technologies.

We sincerely hope that this Special Issue will provide valuable insights and inspire further interest in ongoing efforts to enhance our understanding of the prevention, diagnosis, and treatment of eye diseases. Our objective is that readers find these papers interesting, stimulating, and thought-provoking. Also, we are actively collecting papers for a future Special Issue that will explore further advancements in understanding ocular pathology and its connections to various systemic conditions, titled “Cellular and Molecular Insights into Ocular Changes Associated with Systemic Disorders and Conditions“ https://www.mdpi.com/journal/ijms/special_issues/RBV5KNFL54. (Accessed on 31 December 2023).
